# Investigation of astrovirus, coronavirus and paramyxovirus co-infections in bats in the western Indian Ocean

**DOI:** 10.1186/s12985-021-01673-2

**Published:** 2021-10-12

**Authors:** Axel O. G. Hoarau, Steven M. Goodman, Dana Al Halabi, Beza Ramasindrazana, Erwan Lagadec, Gildas Le Minter, Marie Köster, Andréa Dos Santos, M. Corrie Schoeman, Eduardo S. Gudo, Patrick Mavingui, Camille Lebarbenchon

**Affiliations:** 1 Processus Infectieux en Milieu Insulaire Tropical, INSERM 1187, CNRS 9192, IRD 249, Université de La Réunion, Sainte-Clotilde, La Réunion France; 2 Association Vahatra, Antananarivo, Madagascar; 3 Field Museum of Natural History, Chicago, USA; 4 Veterinary Faculty, Eduardo Mondlane University, Maputo, Mozambique; 5 School of Life Sciences, University of Kwa-Zulu Natal, Kwa-Zulu Natal, South Africa; 6 Instituto Nacional de Saúde, Maputo, Mozambique; 7 Present Address: Institut Pasteur de Madagascar, Antananarivo 101, BP 1274, Ambatofotsikely, Madagascar

**Keywords:** Madagascar, Mozambique, *Triaenops menamena*, *Triaenops afer*, Multiple infections

## Abstract

Co-infections have a key role in virus transmission in wild reservoir hosts. We investigated the simultaneous presence of astroviruses, coronaviruses, and paramyxoviruses in bats from Madagascar, Mayotte, Mozambique, and Reunion Island. A total of 871 samples from 28 bat species representing 8 families were tested by polymerase chain reactions (PCRs) targeting the RNA-dependent RNA-polymerase genes. Overall, 2.4% of bats tested positive for the presence of at least two viruses, only on Madagascar and in Mozambique. Significant variation in the proportion of co-infections was detected among bat species, and some combinations of co-infection were more common than others. Our findings support that co-infections of the three targeted viruses occur in bats in the western Indian Ocean region, although further studies are needed to assess their epidemiological consequences.

**Supplementary Information:**

The online version contains supplementary material available at 10.1186/s12985-021-01673-2.

Co-infection (sometimes written as coinfection) can be defined as the simultaneous infection by at least two genetically different infectious agents in the same host [1–5]. It can affect both host fitness and disease transmission dynamics, therefore playing a critical role in the epidemiology of infectious agents [4–7]. In bats, although many studies have focused on the detection of emerging viruses such as astroviruses (AstVs), coronaviruses (CoVs), and paramyxoviruses (PMVs), limited data is available regarding co-infection patterns and its potential effect on host fitness and disease transmission dynamics [8–13].

In previous studies, we have investigated the presence of either AstVs, CoVs or PMVs in bats in the western Indian Ocean (WIO) region [14–23]. Here, we conducted a meta-analysis of published data [19–21, 23] and performed additional molecular screening in order to obtain a final dataset of 871 bat samples tested for AstVs, CoVs, and PMVs from 28 species representing 8 bat families (see Additional file 1 and Additional file 2).

Biological material was collected on Madagascar, in Mozambique, on Mayotte, and on Reunion Island as part of previous investigations on infectious agents circulation in bats (details relating to the collection of biological material are available in [23]). The list of samples included in this study (e.g. bat species, location, date, type of samples) is provided in the Additional file 1. All samples were previously tested for the presence of CoV [23]; some of them were also tested for the presence of AstV (516 samples; [20, 21]) and PMV (167 samples; [19]). Additional assays were thus performed for the detection of the AstV (355 samples) and PMV (704 samples) RNA-dependent RNA-polymerase (RdRp) genes. Molecular detection was performed using semi-nested polymerase chain reactions (PCRs), as previously described [16, 17, 19–21, 24, 25]. PCR products were visualized on 2% agarose gels stained with 2% Gelred (Biotium, Hayward, CA, USA). Pearson Chi square tests were conducted to examine the effect of the roost sites (i.e. cave, building, tree), host species, sex, and sampling location (i.e. country or island), on virus detection, and to investigate potential associations between AstV, CoV, and PMV. Analyses were conducted with R, version 4.0.5 [26].

PCR products of expected size were submitted for direct Sanger sequencing (Genoscreen, Lille, France). Nucleotide sequences were aligned to generate consensus sequences, and were edited manually using ChromasLite 2.6.5 (Technelysium Pty, South Brisbane, Australia). The 33 partial AstV sequences and 13 partial PMV sequences generated in this study were deposited in GenBank respectively under the accession numbers MZ614404 to MZ614436 and MZ614437 to MZ614449. Genetic diversity was explored with pairwise distance values obtained from *phangorn* package in R, version 2.6.3 [27]. Sequences were compared to reference sequences in NCBI GenBank using the Basic Local Alignment Search Tool (BLAST) with the standard nucleotide BLAST (BLASTn) algorithm (BLAST was performed on August 18th, 2021) [28, 29]. Then, AstV and PMV sequences generated in this study were respectively aligned with 105 and 74 reference partial nucleotide sequences, using CLC Sequence Viewer version 7.6.1 (CLC Bio, Aarhus, Denmark). Phylogenetic trees were generated by maximum-likelihood using PhyML software 3.1 [30], with a GTR evolutionary model, and 1000 bootstrap replicates.

One hundred and forty-two samples tested positive for AstV (mean detection rate ± 95% confidence interval: 16.3% ± 2.5%) (Additional file 2). Positive samples were detected only in Mozambique (20.2% ± 3.6%) and on Madagascar (18.6% ± 3.5%), without significant variation between these two locations (χ^2^ = 0.3, df = 1, *P* > 0.5). Significant differences in AstV detection were observed between roost types (χ^2^ = 6.2, df = 1, *P* < 0.05), between species (χ^2^ = 311.8, df = 24, *P* < 0.001) and between males (20.9% ± 3.8%) and females (11.6% ± 3.0%) (χ^2^ = 5.9, df = 1, *P* < 0.05). AstV prevalence was significantly higher in bats species roosting in caves (27.5% ± 3.9%) than in buildings (2.5% ± 2.0%). On Madagascar, a high detection rate was found in species of the genus *Miniopterus* as compared to the other taxa (χ^2^ = 162.4, df = 1, *P* < 0.05), and especially in *Miniopterus manavi* (88.9% ± 14.5%), *Miniopterus gleni* (86.7% ± 17.2%), and *Miniopterus sororculus* (71.4% ± 33.5%). The highest AstV detection rate in Mozambique was observed in *Triaenops afer* (68.6% ± 12.7%).

A total of 32 samples tested positive for PMV (3.7% ± 1.3%) (Additional file 2). Positive samples were detected only on Madagascar (5.2% ± 1.3%) and in Mozambique (2.7% ± 1.1%), without significant difference between locations (χ^2^ = 2.5, df = 1, *P* > 0.5). Significant differences were detected between males (5.4% ± 2.1%) and females (1.9% ± 1.3%) (χ^2^ = 7.6, df = 1, *P* < 0.05), and between roost sites (χ^2^ = 7.3, df = 2, *P* < 0.01). Bats living in caves were more frequently positive (5.7% ± 2.0%) than those living in buildings (1.7% ± 1.6%). The proportion of positive bats was also significantly different between species on Madagascar (χ^2^ = 91.9, df = 27, *P* < 0.001), particularly in *Triaenops menamena* (42.4% ± 16.9%). In Mozambique, differences between species were not statistically significant (χ^2^ = 1.3, df = 3, *P* > 0.05).

The detection of CoV was conducted as part of a previous study [23]. Briefly, 82 of the 871 samples were positive for CoV (9.4% ± 1.9%), with a higher prevalence in Mozambique (20.5% ± 2.7%) (χ^2^ = 50.4, df = 3, *P* < 0.001) and no difference between males and females (χ^2^ = 2.7, df = 1, *P* > 0.05) (Additional file 2). Positive samples were detected only in bats roosting in caves (10.7% ± 2.6%) and buildings (8.0% ± 3.1%), without statistical difference between these two roost sites (χ^2^ = 1.7, df = 1, *P* > 0.05). However, a significant variation was detected among bat species (χ^2^ = 125.7, df = 27, *P* < 0.001). In Mozambique, the highest prevalence was detected in the cave roosting *Rhinolophus lobatus* (66.7% ± 30.8%). No difference was observed among the species that tested positive on Madagascar (χ^2^ = 2.3, df = 3, *P* > 0.05).

The overall proportion of positive bats detected for either AstVs or PMVs was consistent with previous studies performed in the WIO region, and in other tropical regions [31–34]. Interestingly, higher detection rates were found for both viruses in bats using caves as day-roost sites, suggesting that cave-roosting behavior maybe favorable for horizontal transmission between bats [35]. Differences between locations, sex, and bat species may be explained by a range of factors. For example, seasonality has been identified as a major driver of the infection dynamics of many pathogens, affecting both host susceptibility and transmission [17, 36–39]. Important seasonal variation in the prevalence of infected animals can depend on the period the samples were collected, and, in turn, can lead to misrepresentative conclusions regarding the level of bat exposure to viruses, in particular in cross-sectional studies. Longitudinal studies in wild animals are thus important to precisely assess prevalence of infected animals and its temporal variation [17, 37, 40].

Twenty-one of the 871 samples tested positive for more than one virus (2.4% ± 1.0%). These co-infections were detected only in Mozambique (5.0% ± 2.7%) and on Madagascar (1.7% ± 1.1%), with significant variation among these locations (χ^2^ = 7.0, df = 1, *P* < 0.01) (Fig. 1, Additional file 3). In both location, co-infections were detected only in bats roosting in cave (Madagascar: χ^2^ = 6.4, df = 1, *P* < 0.05; Mozambique: χ^2^ = 1.9, df = 1, *P* > 0.05), and no significant variation was observed between males and females (Madagascar: χ^2^ = 3.6, df = 1, *P* > 0.05; Mozambique: χ^2^ = 3.3, df = 1, *P* > 0.05). Globally, co-infections involving AstV-CoV (1.4% ± 0.8%) and AstV-PMV (1.1% ± 0.7%) were more frequently detected than CoV-PMV (0.1% ± 0.3%) and AstV-CoV-PMV (0.3% ± 0.4%) (χ^2^ = 11.3, df = 3, *P* < 0.05) (Fig. 1). The presence of AstVs was strongly correlated to the presence of PMVs (χ^2^ = 4.6, df = 1, *P* < 0.05) suggesting a potential positive association between these two viruses. Co-infections involving AstVs, CoVs or PMVs were reported in other tropical bat species. For example, a study of *Hipposideros cervinus* on Borneo reported 4.1% of AstV-CoV coinfected bats with a positive association between these two viruses [11].

**Fig. 1:**
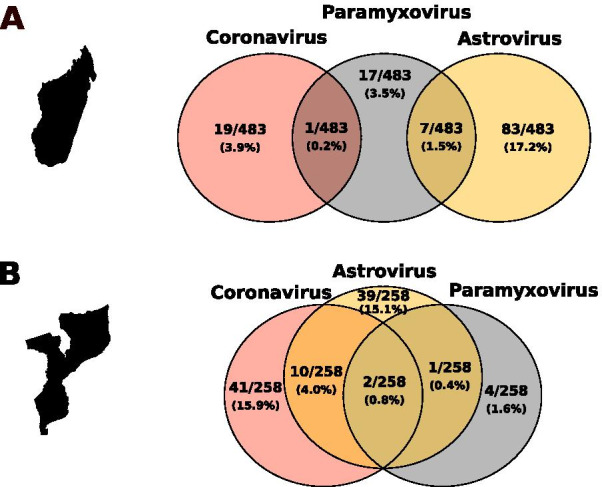
Global co-infection patterns by astroviruses, coronaviruses and pamaxyxoviruses in bats from Madagascar (**A**) and Mozambique (**B**)

On Madagascar, co-infections were detected in three of the 18 tested species: *Triaenops menamena* (15.2% ± 24.7%), *Miniopterus gleni* (13.3% ± 14.8%), and *Paratriaenops furculus* (3.2% ± 0.2%), without significant difference between species (χ^2^ = 2.7, df = 2, *P* > 0.05) (Fig. 2). In Mozambique, co-infections were detected in four of the eight tested species: *Triaenops afer* (15.7% ± 10.0%), *Nycteris thebaica* (7.1% ± 13.5%), *Hipposideros caffer* (5.1% ± 5.6%), and *Miniopterus mossambicus* (4.8% ± 9.1%) (χ^2^ = 4.4, df = 3, *P* > 0.05), also without significant difference between species (Fig. 3). Interestingly, co-infections were detected in bats of the family Rhinonycteridae both in Mozambique (*Triaenops*) and on Madagascar (*Triaenops* and *Paratriaenops*). In Mozambique, the proportion of co-infections was significantly higher in *Triaenops afer* than in all other tested species (χ^2^ = 4.4, df = 1, *P* < 0.05). Also, as compared to other species that tested positive for co-infection, different combinations of co-infections (i.e. different associations between viruses) were detected in species of the genus *Triaenops*. Indeed, *Triaenops menamena* on Madagascar presented two types of co-infections: AstV-PMV and CoV-PMV, whereas *Triaenops afer* in Mozambique harbored three types: AstV-CoV, AstV-PMV, and AstV-CoV-PMV; while other species, including *Paratriaenops furculus*, only presented one co-infection type (Figs. 2 and 3 and Additional file 3). Altogether, these results highlight multiple infections in WIO region bats, and give rise to additional questions concerning variation among species, as well as their consequences on viral infection dynamics.

**Fig. 2:**
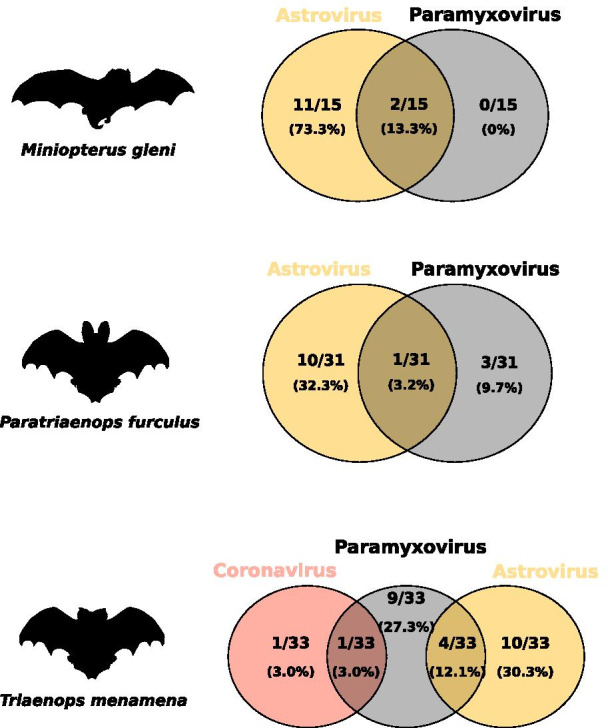
Co-infection by astroviruses, coronaviruses and paramyxoviruses in bat species from Madagascar

**Fig. 3:**
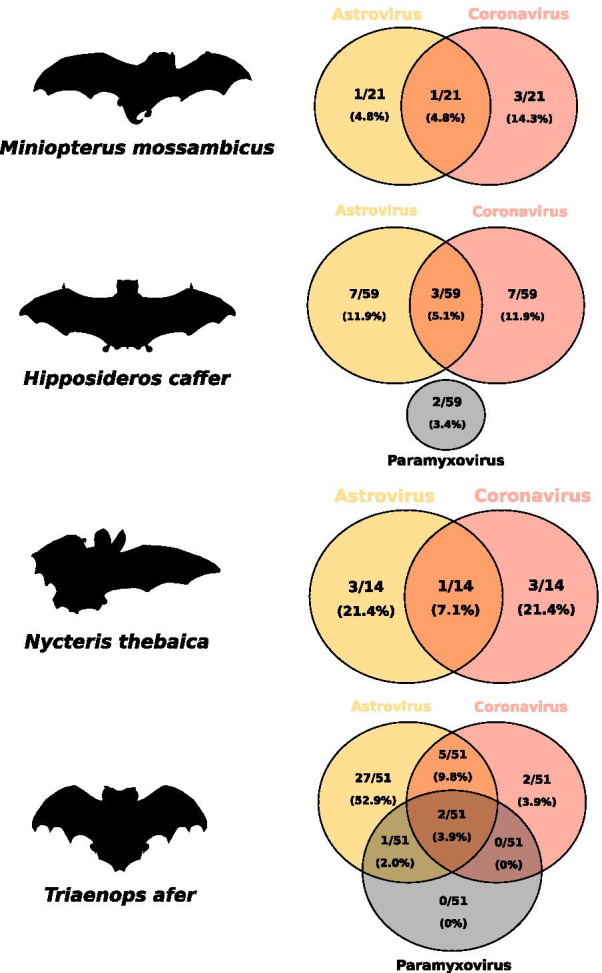
Co-infection by astroviruses, coronaviruses and paramyxoviruses in bat species from Mozambique

High genetic diversity was detected for AstV, with pairwise differences up to 46% between sequences, without support for host family or species association (Fig. 4). Based on BLASTn comparisons, we found that our sequences had a high level of identity (between 80 and 92%) with AstV previously described in bats of the WIO region [20, 21], as well as with AstVs detected in bats from continental Africa (e.g. Gabon, Democratic Republic of Congo), and in other regions in the world (e.g. China, Thailand) (Additional file 4). However, one sequence obtained from a *Triaenops menamena*, a species endemic to Madagascar, showed 92% identity to an AstV sequence detected in a mouse from China (Additional file 4). Another example, even more unexpected, one AstV sequence obtained from *Chaerephon leucogaster* on Madagascar had 96% identity with an AstV sequence from a bird of the order Passeriformes (Additional file 4). These findings were consistent with phylogenetic results and were statistically supported (Fig. 4). A recent study also reported AstVs related to avastrovirus in environmental samples collected in a colony of *Mormopterus francoismoutoui,* a member of the family Molossidae endemic to Reunion Island [41]. These findings may suggest introduction of AstVs on the island by non-native rodents, and could also support environmental transmission of AstVs between species of different taxa, as previously suggested [42]. Nevertheless, studies investigating the circulation of AstVs in terrestrial small mammals in the WIO region are required to assess these potential host-shifts.

**Fig. 4:**
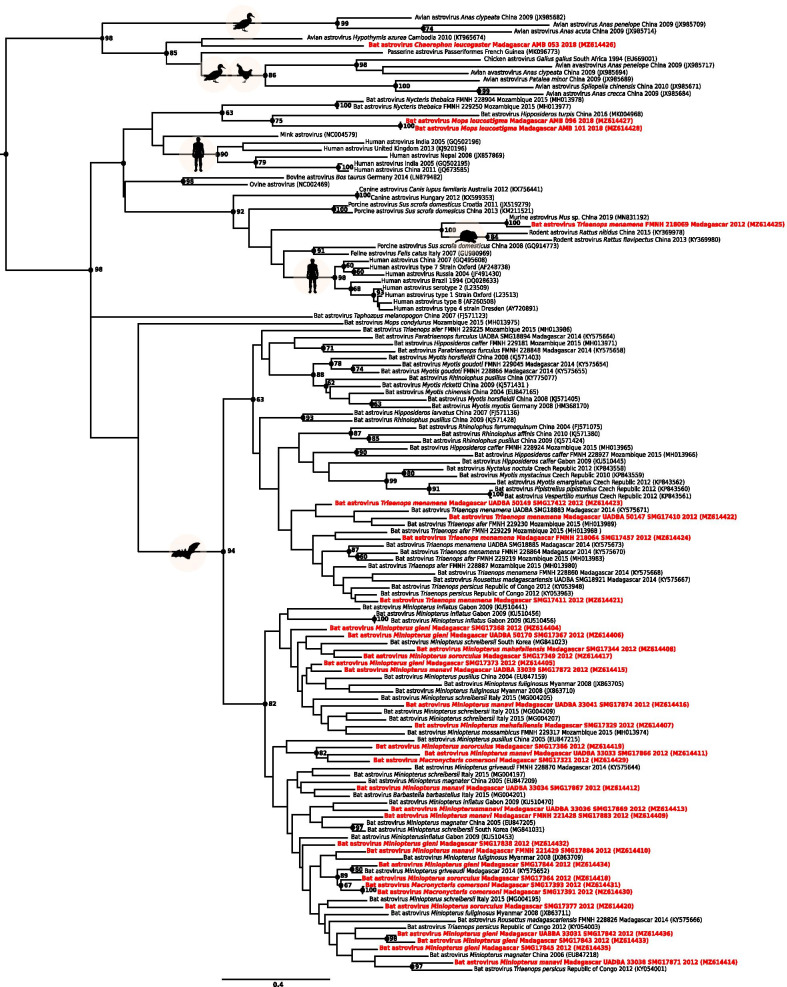
Maximum likelihood consensus tree derived from 138 astrovirus (AstV) RNA-dependent RNA-polymerase partial nucleotide sequences (397 bp). Black dots indicate nodes with bootstrap values higher or equal than 60. Sequence names colored in red indicate bat AstVs detected in this study. Sequence accession numbers are indicated in parentheses

Genetic diversity was less important for PMV sequences, with pairwise differences up to 29%. All our sequences were genetically related to PMVs previously described on Madagascar [16, 19] or in continental Africa (e.g. Ghana, Kenya), with sequence identity ranging from 78 to 99% (Additional file 5 and Fig. 5). Phylogenetic analyses highlighted some degree of host-specificity, as previously described for PMV in the western Indian Ocean (Fig. 5) [16]. For instance, most sequences clustered either with PMV sequences detected in bats on the same genus captured in the region or elsewhere (e.g. *Hipposideros caffer* from Mozambique), or with sequences obtained in bats from the same family (e.g. *Mops condylurus* sequence from Mozambique, and *Chaerephon leucogaster* sequence from Madagascar clustered with a sequence detected in *Mops leucostigma* on Madagascar). However, some sequences were included in more diversified groups including different bat families (e.g. two sequences obtained from *Paratriaenops furculus* on Madagascar that clustered with sequences detected in *Miniopterus griveaudi* and *Chaerephon leucogaster* on Madagascar).

**Fig. 5:**
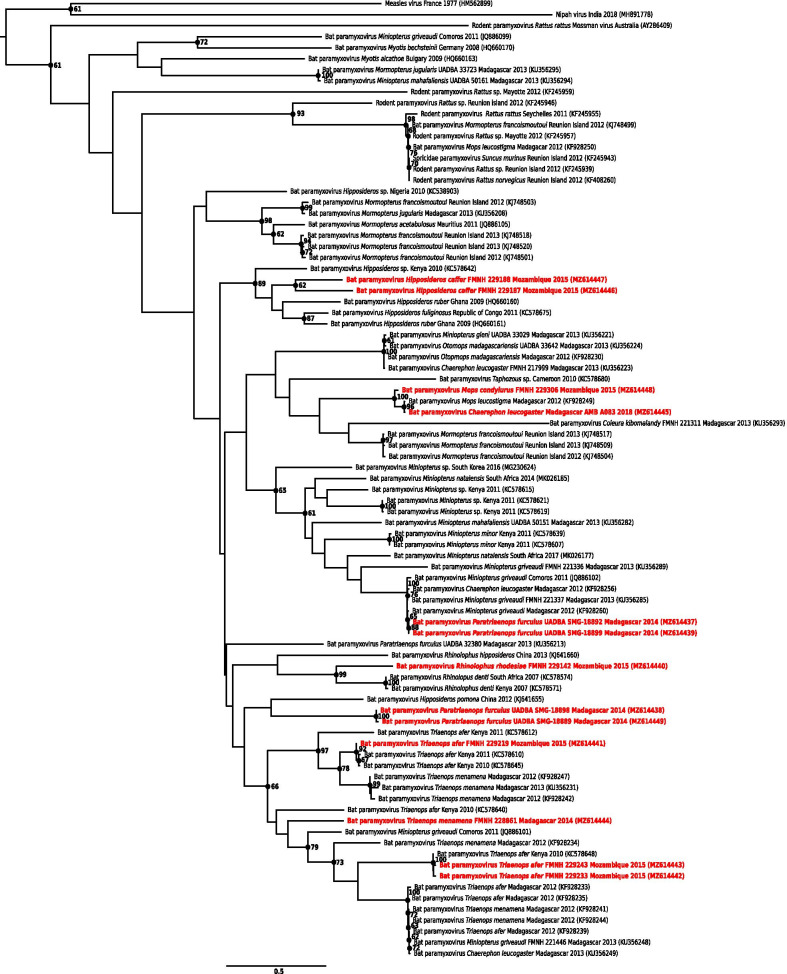
Maximum likelihood consensus tree derived from 87 paramyxovirus (PMV) RNA-dependent RNA-polymerase partial nucleotide sequences (439 bp). Black dots indicate nodes with bootstrap values higher or equal than 60. Sequence names colored in red indicate bat PMVs detected in this study. Sequence accession numbers are indicated in parentheses

We report co-infections in bats on Madagascar and in Mozambique, ranging from 3.2% to 15.7% of the positive samples, and depending on the tested bat species. Although our cross-sectional sampling precludes detailed interpretation of the biological drivers of such variation, our results nevertheless highlight that interactions between infectious agents in bats may exist with potential consequences on their epidemiology. AstVs, CoVs, and PMVs are emerging viruses that represent a major challenge for human and animal health. Further knowledge on virus interaction in wildlife, based on long-term longitudinal sampling is needed to fully assess the epidemiological consequences of co-infections [5].

## Supplementary Information

**Additional file 1**. List and origin of samples, day roost sites, and result of the molecular screening.

**Additional file 2**. Number of tested and positive samples, per location, bat family, species, samples types, and collection year.

**Additional file 3**. Number of tested and positive samples for co-infections, per country, bat family, bat species, sample type, and collection year.

**Additional file 4**. Astrovirus nucleotide sequence similarity obtained with BLASTn.

**Additional file 5**. Paramyxovirus nucleotide sequence similarity obtained with BLASTn

## Data Availability

Data are available in the supplementary material (detailed list of samples and results of molecular screening) and in GenBank (sequence accession numbers of the sequences generated in this study: MZ614404 to MZ614449).

## Abbreviations

AstV: Astrovirus
BLAST: Basic local alignment search tool
BLASTn: Standard nucleotide BLAST
CoV: Coronavirus
PCR: Polymerase chain reaction
PMV: Paramyxovirus
RdRp: RNA-dependent RNA-polymerase
WIO: Western Indian Ocean
